# TGFβ1 signaling sustains aryl hydrocarbon receptor (AHR) expression and restrains the pathogenic potential of T_H_17 cells by an AHR-independent mechanism

**DOI:** 10.1038/s41419-018-1107-7

**Published:** 2018-11-13

**Authors:** Kalil Alves de Lima, Paula Barbim Donate, Jhimmy Talbot, Marcela Davoli-Ferreira, Raphael Sanches Peres, Thiago Mattar Cunha, José Carlos Alves-Filho, Fernando Queiroz Cunha

**Affiliations:** Inflammation and Pain Laboratory, Center for Research in Inflammatory Diseases, Ribeirao Preto Medical School, University of Sao Paulo, Ribeirao Preto, Sao Paulo, Brazil

## Abstract

The aryl hydrocarbon receptor (AHR) is a transcription factor activated by ligand highly expressed on T_H_17 cells, and AHR-deficient CD4^+^ T cells have impaired production of IL-17A and IL-22. Although AHR activation can exacerbate in vivo T_H_17 cell-mediated autoimmunity, accumulating data indicate that AHR is a nonpathogenic T_H_17 marker. Thus it remains unclear how AHR activation is regulated and impacts on the generation of T_H_17 subsets. Here we demonstrated that AHR pathway is activated during in vitro pathogenic T_H_17 polarization, but it is quickly downregulated. Under these conditions, additional AHR activation promoted IL-22 but not IL-17A. Interestingly, AHR high sustained expression and IL-17A promotion were only achieved when TGFβ1 was present in the culture. In addition to the effect on AHR regulation, TGFβ1 presented a dual role by simultaneously suppressing the T_H_17 pathogenic phenotype acquisition. This latter effect was independent of AHR stimulation, since its activation did not confer a T_H_17 anti-inflammatory profile and *Ahr*^−^^/−^ cells did not upregulate any T_H_17 pathogenic marker. Through the use of EAE model, we demonstrated that AHR is still functional in encephalitogenic CD4^+^ T cells and the adoptive transfer of *Ahr*^−^^/−^ T_H_17 cells to recipient mice resulted in milder EAE development when compared to their WT counterparts. Altogether, our data demonstrated that although AHR is highly expressed on in vitro-generated nonpathogenic T_H_17 cells, its ligation does not shift T_H_17 cells to an anti-inflammatory phenotype. Further studies investigating the role of AHR beyond T_H_17 differentiation may provide a useful understanding of the physiopathology of autoimmune diseases.

## Introduction

T helper type 17 (T_H_17) cells are characterized by the production of interleukin-17A (IL-17A), IL-17F, and IL-22 and have emerged as a subset of effector CD4^+^ T cells with an important role in the control of specific pathogens as well as in the development of autoimmune diseases, including rheumatoid arthritis and multiple sclerosis^1,2^. T_H_17 cell differentiation is classically driven by the cytokines transforming growth factor-β1 (TGFβ1) and IL-6, which induce retinoic acid–related orphan receptor γt (RORγt), the lineage-defining transcription factor for T_H_17 cells^3–6^. Additionally, other transcription factors, such as the aryl hydrocarbon receptor (AHR), the interferon regulatory factor 4 (IRF4), and the basic leucine zipper ATF-like transcription factor (BATF), participate in T_H_17 polarization^7–9^.

AHR is highly expressed on T_H_17 cells and has an important role in the in vivo and in vitro generation of these cells^10,11^. Although CD4^+^ T cells from AHR-deficient mice can differentiate into T_H_17 cells, they have impaired production of IL-17A and undetectable IL-22 production^11^. On the other hand, AHR activation under T_H_17 cell-inducing conditions by 6-formylindolo[3,2-b]carbazole (FICZ), a tryptophan-derived photoproduct with high affinity for the AHR receptor, strongly enhances IL-17A production and the percentage of IL-22-producing cells^11–13^. Additionally, AHR activation by FICZ can exacerbate in vivo T_H_17 cell-mediated autoimmunity, such as experimental autoimmune encephalomyelitis (EAE) and collagen-induced arthritis^11,12,14^. Although T_H_17 cells are thought to be pathogenic, accumulating data indicate the existence of in vitro-generated nonpathogenic IL-17A-producing T_H_17 cells^15–20^. Pathogenic and nonpathogenic T_H_17 cell nomenclature was recently suggested through the use of the EAE passive model, since transfer of (1) TGFβ1, IL-6, and IL-23; (2) IL-1β, IL-6, and IL-23; or (3) TGFβ3 and IL-6 differentiated MOG-specific CD4^+^ T cells led to the development of severe disease in mice, whereas mild or no disease was observed when TGFβ1 and IL-6-differentiated T cells were transferred^15^. For this reason, the classically TGFβ1 and IL-6 in vitro-generated T_H_17 cells are described as nonpathogenic T_H_17 cells. Intriguingly, AHR has been described as a nonpathogenic T_H_17 marker, and under specific conditions, its activation induces regulatory T cell polarization^12,21–24^. Thus it remains unclear how AHR is regulated during in vitro pathogenic T_H_17 cell generation and how its modulation impacts on the generation of these subpopulations of T_H_17 cells.

In this study, we characterized the AHR expression pattern of in vitro-generated pathogenic and nonpathogenic T_H_17 cells, and we demonstrated that the AHR pathway is expressed and activated during pathogenic cell polarization, but it is rapidly downregulated with the absence of TGFβ1 in the cocktail. TGFβ1 signaling had a dual role by inducing high AHR expression and consequently more T_H_17 cell differentiation, but it also inhibited T_H_17 pathogenic phenotype acquisition, which was mediated by AHR-independent mechanisms. Consistent with the AHR participation in both in vitro and in vivo T_H_17 cell generation, we demonstrated that AHR is still highly expressed and functional in pathogenic central nervous system (CNS)-infiltrating CD4^+^ T cells, and most importantly, the adoptive transfer of *Ahr*^−^^/−^ MOG-specific fully differentiated T_H_17 cells to recipient *Rag1*^−^^/−^ mice resulted in milder EAE development when compared to their WT counterparts. Taken together, our study demonstrates that, although AHR is highly expressed on in vitro-generated nonpathogenic T_H_17 cells, its activation per se is not responsible for shifting T_H_17 cells to an anti-inflammatory phenotype. Further studies investigating the mechanisms by which AHR mediates a potential pathogenicity in T_H_17 cells may provide a useful understanding of the physiopathology of autoimmune diseases.

## Materials and methods

### Mice

C57BL/6 wild-type (WT), *Ahr*^−^^/−^, and *Rag1*^−^^/−^ (purchased from Jackson Laboratory) mice were housed and maintained in a conventional pathogen-free facility at Ribeirao Preto Medical School, University of Sao Paulo. All mice received water and food ad libitum. All the experiments received approval and were performed in accordance to the guidelines outlined by the Standing Committee on Animals at Ribeirao Preto Medical School, University of Sao Paulo.

### In vitro T cell differentiation

CD4^+^ T cells were purified from spleen and lymph nodes (LNs) with anti-CD4 microbeads (Miltenyi Biotech) and then further sorted as naive CD4^+^CD44^lo^CD62L^hi^ T cells using a FACSAria III sorter (BD Biosciences). Sorted cells were activated with plate-bound anti-CD3 (4 μg/mL) and soluble anti-CD28 (2 μg/mL, both BD Biosciences) in the presence of polarizing cytokines. For T_H_17 differentiation, the following reagents were used: 2.5 ng/mL recombinant human TGFβ1 (eBioscience) and 20 ng/mL recombinant mouse IL-6 (R&D Systems) for nonpathogenic T_H_17 cells. Pathogenic T_H_17 cells were differentiated with 25 ng/mL of IL-1β (R&D Systems), 20 ng/mL of IL-6, and 20 ng/mL of IL-23 (R&D Systems). Alternatively, as indicated in the text, TGFβ1-induced pathogenic T_H_17 cells were differentiated in the presence of 2.5 ng/mL TGFβ1, 20 ng/mL IL-6, and 20 ng/mL of IL-23. TGFβ3-induced cells were generated with 2.5 ng/mL TGFβ3 (R&D Systems) and 20 ng/mL IL-6. Cells were cultured for 3 days and collected for RNA, intracellular cytokine staining, and flow cytometry. FICZ (Enzo Life Sciences; 100 nM) and CH223191 (Sigma-Aldrich; 30 μM) were added at the start of the cultures where indicated.

### Flow cytometric analysis

Cells were stimulated for 4 h with phorbol 12-myristate 13-acetate (50 ng/mL; Sigma-Aldrich), ionomycin (500 ng/mL; Sigma-Aldrich), and a protein-transport inhibitor containing Brefeldin (1.5 µL/mL GolgiPlug; BD Biosciences) before detection by staining with antibodies. Surface markers were stained for 10 min at room temperature, then were fixed in Cytoperm/Cytofix (BD Biosciences), permeabilized with Perm/Wash Buffer (BD Biosciences), and stained with intracellular antibodies diluted in Perm/Wash buffer. Data were collected with a FACSVerse or FACSCanto II (BD Biosciences) and then were analyzed with the FlowJo 10 software (Treestar).

### RNA isolation and real-time PCR

RNA was extracted using a PureLink® RNA Mini Kit (Life Technologies), reverse-transcribed with a High Capacity Kit (Life Technologies), and analyzed by quantitative reverse transcriptase-PCR into a Step One Real-time PCR system (Applied Biosystems). The comparative threshold cycle method and an internal control (*Gapdh*) were used for normalization of the target genes. The probes used were identified by the following Applied Biosystems assay numbers: *Ahr (Mm00478932_m1)*, *Ahrr (Mm01352370_m1)*, *Csf2 (Mm01290062_m1)*, *Cyp1a1 (Mm00487218_m1)*, *Il10 (Mm01288386_m1)*, *Il17a (Mm00439618_m1)*, *Il22 (Mm01226722_g1)*, *Il23r (Mm00519943_m1)*, *Gapdh (Mm99999915_g1)*, *Maf (Mm02581355_s1)*, *Rorc (Mm01261022_m1)*, and *Tbx21 (Mm00450960_m1)*.

### Measurement of cytokines

The culture supernatants were harvested and the concentrations of IL-22 (eBioscience) and IL-17A (R&D Systems) were determined by enzyme-linked immunosorbent assay according to the manufacturer’s instructions.

### Active and passive EAE induction

For active induction of EAE, mice were immunized by subcutaneous injection of 200 μg MOG_35–55_ (MEVGWYRSPFSRVVHLYRNGK) in Complete Freund’s Adjuvant (CFA; Sigma Aldrich) and received 200 ng of pertussis toxin intraperitoneally on days 0 and 2 (Sigma-Aldrich). For the passive induction, CD4^+^ cells were positively selected from draining LNs (dLNs) of WT and *Ahr*^−^^/−^ mice immunized with MOG-CFA. These cells were co-cultured with fluorescence-activated cell sorter (FACS)-sorted CD11c^+^ dendritic cells (DCs) isolated from WT immunized mice (1:10, DC to CD4^+^ cells) plus 50 µg/mL MOG (35–55) and 10 ng/mL of IL-23 to skew cells toward a pathogenic T_H_17 phenotype. Differentiation status was checked on day 4 by intracellular cytokine staining and 2.5 × 10^5^ IL-17A-positive T cells were transferred intravenously into *Rag1*^−^^/−^ recipient mice (*n* = 8 mice/group). Recipient mice were also given 200 ng of pertussis toxin intraperitoneally on days 0 and 2. Mice were monitored and assigned scores daily for the development of classical and atypical signs of EAE according to the following criteria: 0, no disease; 1, decreased tail tone or mild balance defects; 2, hind limb weakness, partial paralysis, or severe balance defects that cause spontaneous falling over; 3, complete hind limb paralysis or very severe balance defects that prevent walking; 4, front and hind limb paralysis or inability to move body weight into a different position; 5, moribund state.

### Isolation of CNS-infiltrating cells

Mice with scores 2–3 were sacrificed and perfused through the left ventricle of the heart with cold phosphate-buffered saline. The spinal cord was minced with a sharp razor blade and digested for 45 min at 37 °C with collagenase D (2.5 mg/mL; Roche Diagnostics). Mononuclear cells were isolated by passage of the tissue through a cell strainer (40 μm), followed by centrifugation through a Percoll gradient (37% and 70%). Mononuclear cells in the interphase were removed, washed, and resuspended in culture medium for further analysis.

### Statistical analysis

GraphPad Prism 6.0 was used for statistical analysis (unpaired, two-tailed Student’s *t* test or two-way analysis of variance). Differences were considered statistically significant with a *P* value of <0.05.

## Results

### Differential AHR expression during in vitro pathogenic and nonpathogenic T_H_17 cell differentiation

It is well known that AHR activation mediates IL-22 production in T_H_17 cells both in vitro and in vivo^10,11^. Accordingly, incubation of CD4^+^CD44^lo^CD62L^hi^ naive T cells with T_H_17 differentiation cocktail (TGFβ1 and IL-6) plus an AHR agonist (FICZ) increased T_H_17 cell differentiation, as seen by higher IL-17A^+^ and IL-22^+^ cell frequency and IL-22 secretion into the culture supernatants. On the other hand, AHR-deficient naive T CD4^+^ cells, or the pharmacological inhibition of AHR with CH223191, impaired the polarization of T_H_17 cells producing both IL-17A and IL-22 and stopped the secretion of IL-22 (Supplemental Figs. 1A, 1B and 1C).

**Fig. 1:**
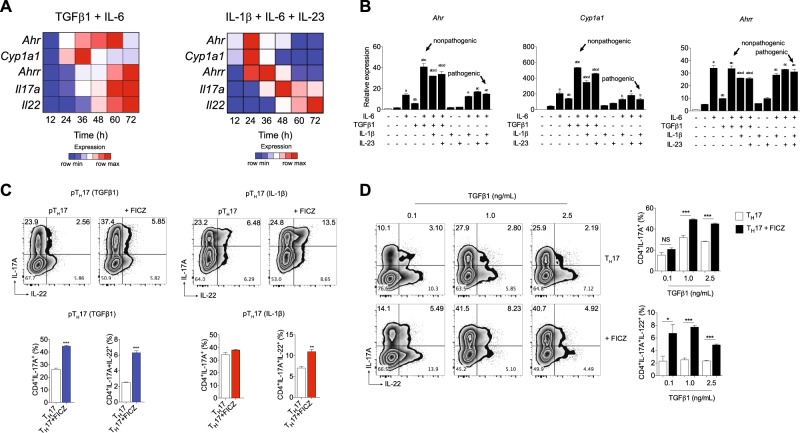
AHR is activated during in vitro pathogenic T_H_17 cell differentiation, and it regulates IL-17A production in a TGFβ1-dependent manner. **a** Heatmap of *Ahr*, *Cyp1a1*, *Ahrr*, *Il22*, and *Il17a* mRNA expression in CD4^+^CD44^lo^CD62L^hi^ naive T cells differentiated for 12, 24, 36, 48, 60, and 72 h under nonpathogenic (TGFβ1 plus IL-6) and pathogenic (IL-1β, IL-6, and IL-23) T_H_17 conditions. **b** qPCR analysis of *Ahr*, *Cyp1a1*, and *Ahrr* mRNA expression of naive CD4^+^ T cells (white bars) activated 24 h under different combinations of IL-6, TGFβ1, IL-1β, and IL-23 (as indicated). **c** Frequency of IL-17A^+^ and IL-22^+^ cells from pathogenic T_H_17 cells differentiated with TGFβ1, IL-6 and IL-23 (pT_H_17 (TGFβ1)) or IL-1β, IL-6 plus IL-23 (pT_H_17 (IL-1β)) in the presence of FICZ. **d** Frequency of IL-17A^+^ and IL-22^+^ cells from pT_H_17 cells (TGFβ1, IL-6, and IL-23) differentiated under different concentrations of TGFβ1. **b** *P* < 0.05 when compared to ^a^medium; ^b^IL-6; ^c^TGFβ1, and ^d^TGFβ1+IL-6 groups (two-way ANOVA). NS not significant; *P < 0.05, *P *<* 0.01, and ***P *<* 0.001 (**c**, unpaired, two-tailed Student’s *t* test and **d** two-way ANOVA). Data are representative of more than three independent experiments with similar results

Although IL-22 is highly expressed in pathogenic T_H_17 cells, it has been described that these cells have modest AHR expression compared to nonpathogenic T_H_17 cells^15,16^ (Supplemental Fig. 1D). To gain a better understanding of these mechanisms, we investigated whether the AHR pathway is differently activated during in vitro pathogenic and nonpathogenic T_H_17 cell polarization. Pathogenic and nonpathogenic cells were differentiated using IL-1β+IL-6+IL-23 or TGFβ1 plus IL-6, respectively, and the kinetic of AHR-regulated genes was analyzed by quantitative PCR (qPCR). AHR gene expression was upregulated significantly from 12 h after the start of T_H_17 differentiation, and its expression remained high throughout the nonpathogenic T_H_17 differentiation (Fig. 1a and Supplemental Table 1). Of note, the upregulation of *Ahr* transcripts was followed by the expression of the gene reporter of AHR activation, *Cyp1a1*, which supports the endogenous AHR stimulation. The AHR repressor (AHRR) gene (encoded by *Ahrr*) appeared in the late stage of nonpathogenic T_H_17 induction (Fig. 1a and Supplemental Table 1). In contrast to nonpathogenic T_H_17 cells and, although we also have observed induction of *Ahr* and *Cyp1a1*, these genes were quickly downregulated about 36–48 h after the start of pathogenic cell polarization (Fig. 1a and Supplemental Table 1). Additionally, *Il22/Il17a* genes had the highest expression during the late time points of the differentiation. These results demonstrate that the AHR pathway is activated during both pathogenic and nonpathogenic T_H_17 cell differentiation; however, during pathogenic T_H_17 conditions, its expression is quickly downregulated. Interestingly, when TGFβ3 was used in the cocktail to induce pathogenic T_H_17 cell differentiation, the AHR kinetic pathway was comparable to IL-1β-induced pathogenic cells (Supplemental Fig. 2A and Supplemental Table 1).

### Optimal AHR expression and IL-17A promotion by AHR activation are TGFβ1 dependent

We next addressed whether *Ahr* expression in pathogenic and nonpathogenic T_H_17 cells occurred at the same intensity when different cytokine cocktails were used. Although TGFβ1 or IL-6 alone induced modest *Ahr* expression, we observed a significant synergism between these cytokines to drive the highest AHR expression (Fig. 1b). Of note, IL-23 and IL-1β addition had a slight negative effect of regulating its expression by T cells. Moreover, *Cyp1a1* expression was correlated with the *Ahr* pattern, and at this early time point (24 h), the AHRR transcription was similar between the conditions containing IL-6 (Fig. 1b). Considering that TGFβ3 signaling also drives pathogenic T_H_17 cell generation^15^, we did not find high *Ahr* induction in the presence of TGFβ3 plus IL-6 (Supplemental Fig. 2B). Altogether, these data demonstrated that *Ahr* is weakly induced under pathogenic T_H_17-polarizing conditions, and its optimal and sustained expression is driven by IL-6 in combination with TGFβ1 (but not TGFβ3).

To assess whether AHR is still functional in pathogenic T_H_17 cells, we determined IL-22 and IL-17A promotion in pathogenic cells differentiated upon FICZ stimulation. On the basis of our previous data showing TGFβ1 dependence to drive high *Ahr* expression, pathogenic T_H_17 cells were polarized with IL-1β, IL-6, and IL-23 or TGFβ1, IL-6, and IL-23. Consistent with the kinetics observation during pathogenic differentiation programs, FICZ stimulation significantly increased the frequency of IL-17A^+^IL-22^+^ cells (Fig. 1c), IL-22 gene expression and cytokine secretion (Supplemental Figs. 1E and 1F). The same result was also obtained using pathogenic T_H_17 cells differentiated by TGFβ3 and IL-6 (Supplemental Fig. 2b). Interestingly, no significant IL-17A promotion was observed in pathogenic T_H_17 cells polarized in the absence of exogenous TGFβ1, and the effects of TGFβ1 were dose dependent (Fig. 1c, d and Supp. Figure 2C and D). These data suggested that AHR signaling is still functional during pathogenic T_H_17 cell differentiation and highlight a pivotal role of TGFβ1 signaling by inducing high levels of AHR expression and consequently IL-17A promotion.

### TGFβ1 maintains AHR expression while opposing IL-23-driven pathogenic conversion of in vitro-generated T_H_17 cells

Pathogenic T_H_17 cells induced by a combination of IL-1β, IL-6 plus IL-23 or TGFβ3 plus IL-6 had transient AHR expression. T_H_17 cells show a high degree of developmental flexibility, and when exposed to IL-23, they rapidly shift to a T_H_1 cell-like phenotype frequently associated with pathogenic effector functions^25–29^. Based on the well-known negative effect of TGFβ1 signaling in the encephalitogenic properties of T_H_17 cells, we hypothesized that TGFβ1 in the cultures may lead to a misinterpretation that AHR activation is responsible to drive anti-inflammatory functions to T cells. In order to address this hypothesis, we examined the acquisition of pathogenicity markers and the AHR responsiveness of T_H_17 cells generated and propagated under defined cytokine conditions in vitro.

**Fig. 2:**
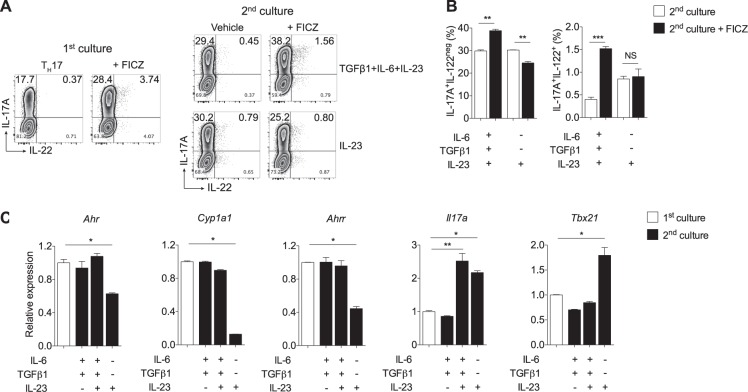
TGFβ1 signaling maintains *Ahr* expression in T_H_17 cells, while it opposes IL-23-driven conversion into pathogenic T_H_17 cells. **a** Naive CD4^+^ T cells were cultured 3 days under nonpathogenic T_H_17-polarizing conditions (TGFβ1+IL-6) in the absence or presence of FICZ and then stained intracellularly for IL-17A and IL-22 (first culture). A fraction of recovered cells cultured without FICZ was re-stimulated for additional 3 days with TGFβ1, IL-6 plus IL-23, or only IL-23, with or without FICZ stimulation (second culture). **b** Frequencies of IL-17A^+^ and IL-22^+^ cells after the second culture shown in **a**. **c** qPCR analysis of *Ahr*, *Cyp1a1*, *Ahrr*, *Il17a*, and *Tbx21* of naive CD4^+^ T cells cultured under nonpathogenic T_H_17-polarizing conditions (TGFβ1+IL-6) for 3 days (first culture, white bars) and re-stimulated with TGFβ1, IL-6, and IL-23 combinations for 24 h (second culture). NS not significant; *P < 0.05, **P < 0.01, and ***P < 0.001 (two-way ANOVA)

Supporting our previous data that TGFβ1 signaling is crucial to the AHR-dependent IL-17A induction, we observed that nonpathogenic T_H_17 cells propagated during a second culture without exogenous TGFβ1 demonstrated no IL-17A promotion upon AHR activation (Fig. 2a, b). Remarkably, cells cultured with IL-23 alone during this second culture acquired more T_H_1 cell-like phenotype with more interferon-γ (IFN-γ) production (data not shown).

To further correlate these data with the AHR signaling pathway, we re-stimulated fully differentiated nonpathogenic T_H_17 cells in the combination of TGFβ1, IL-6, and IL-23 cytokines for 24 h, and we performed qPCR of AHR-related genes and the pathogenicity T_H_1 cell-like marker, *Tbx21*. Notably, cells maintained only with IL-23 showed *Ahr*, *Cyp1a1*, and *Ahrr* downregulation but *Tbx21* upregulation (Fig. 2c). These data suggest that, while TGFβ1 maintains AHR-related gene expression, its presence restrain IL-23-driven nonpathogenic conversion into pathogenic T_H_17 cells.

### AHR signaling does not confer anti-inflammatory properties to T_H_17 cells

**Fig. 3:**
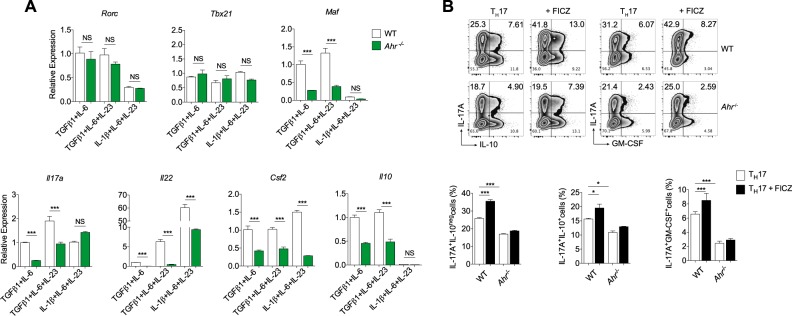
AHR signaling does not drive anti-inflammatory properties to T_H_17 cells. Quantitative RT-PCR analysis of *Rorc*, *Tbx21*, *Maf*, *Il17a*, *Il22*, *Csf2*, and *l10* from WT (white bars) or *Ahr*-deficient (green bars) cells differentiated for 72 h under nonpathogenic (TGFβ1 and IL-6) and pathogenic T_H_17 cell conditions (TGFβ1, IL-6 plus IL-23 or IL-1β, IL-6 and IL-23). **b** Frequency of IL-17A^+^, IL-10^+^, and GM-CSF^+^ cells from WT and *Ahr*^−^^/−^ naive CD4^+^ T cells differentiated for 72 h with TGFβ1+IL-6+IL-23 under FICZ stimulation. NS not significant; *P < 0.05 and ***P < 0.001 (two-way ANOVA)

Even though AHR expression in nonpathogenic T_H_17 cells has been described, its role in regulating preferentially anti-inflammatory molecules in these cells has not been clearly shown. To isolate the indirect effect mediated through TGFβ1 signaling, we investigated whether AHR-deficient T_H_17 cells would attain more pathogenic characteristics. For this purpose, we polarized WT or *Ahr*^−^^/−^ naive CD4^+^ T cells into nonpathogenic and pathogenic T_H_17 cells and compared their expression of the T_H_17 pathogenicity markers. In accordance to previous reports, WT pathogenic T_H_17 cells differentiated by IL-1β, IL-6, and IL-23 were characterized by higher expression of *Il22* and *Csf2* transcripts when compared to T_H_17 cells polarized in the presence of TGFβ1 (Fig. 3a). These cells also showed less expression of genes encoding anti-inflammatory markers, such as *Maf* and *Il10*. Notably, under TGFβ1 signaling stimulation, AHR-deficient T_H_17 cells expressed diminished levels of *Maf* and all cytokines evaluated (*Il17a*, *Il22*, *Csf2*, and *Il10*) (Fig. 3a). The lack of AHR was also related to lower expression of *Csf2* and *Il22* in IL-1β- and TGFβ3-induced pathogenic cells (Fig. 3a and Supplemental Fig. 2E, respectively).

Given the reported results, we further validated the AHR dependence of IL-17A, IL-10, and granulocyte macrophages colony-stimulating factor (GM-CSF) protein expression during TGFβ1-induced pathogenic T_H_17 polarization. Consistent with the qPCR data, FACS analysis supported AHR regulation of both pro- and anti-inflammatory mediators in T_H_17 cells (Fig. 3b). Of note, neither AHR activation nor its deficiency was able to modulate GM-CSF and IL-10 protein levels in pathogenic T_H_17 cells differentiated by IL-1β, IL-6, and IL-23, whereas IL-17A/GM-CSF double positive cells were significantly decreased in TGFβ3-induced pathogenic T_H_17 cells from *Ahr*^−^^/−^ cells (Supplemental Fig. 2F). Altogether, these findings demonstrate that AHR signaling participates as a cytokine regulator in both pathogenic and nonpathogenic cells and do not support AHR signaling as an anti-inflammatory factor for in vitro-differentiated T_H_17 cells.

### AHR is highly expressed and functional in pathogenic CNS-infiltrating CD4^+^ T cells

It remains unknown whether the phenotype observed in vitro occurs during pathogenic conversion of in vivo-generated T_H_17 cells. Transcriptional changes have been reported in cells differentiated in vitro when compared to in vivo-generated T_H_17 cells^27^. Furthermore, CNS-infiltrating T_H_17 cells of EAE mice have high *Ahr* expression^27^. To assess the in vivo relevance of our in vitro findings and to investigate the AHR modulation in in vivo-generated T_H_17 cells, we immunized WT mice with MOG (35–55) emulsified in CFA and evaluated the expression of AHR-related genes in CD4^+^ T cells isolated from the draining LNs (dLNs) and spinal cord of EAE mice.

**Fig. 4:**
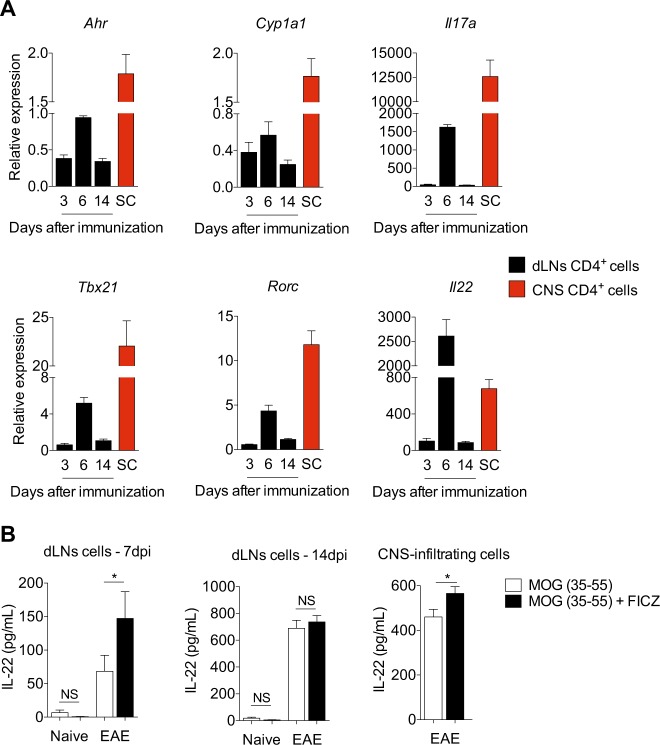
AHR is still highly expressed and functional on CNS-infiltrating pathogenic CD4^+^ T cells. **a** qPCR analysis of *Ahr*, *Cyp1a1*, *Il17a*, *Tbx21*, *Rorc*, and *Il22* transcripts of CD4^+^ T cells isolated from draining lymph nodes (dLNs) after 3, 6, or 14 days after EAE induction. Spinal cord (SC) CD4^+^ T cells were isolated 14 days after MOG immunization. **b** IL-22 production of purified dLNs cells from WT mice 7 and 14 days after EAE induction and CNS-infiltrating cells 14 days after MOG-CFA immunization re-stimulated for 72 h with MOG (50 µg/mL) with or without FICZ (300 nM). NS not significant; *P *<* 0.05 (unpaired, two-tailed Student’s *t* test)

In accordance to AHR participation during disease progression, the *Ahr*/*Cyp1a1* transcripts expression in dLN CD4^+^ T cells was correlated with the expression pattern of other T_H_17 effector markers, such as *Il17a*, *Tbx21*, *Rorc*, and *Il22* (Fig. 4a). Most importantly, high *Ahr*/*Cyp1a1* expression were also observed in CD4^+^ T cells infiltrating the CNS of these mice, demonstrating that AHR signaling pathway is still activated in in vivo pathogenic T CD4^+^ cells (Fig. 4a). Notably, approximately 20% of these CNS-infiltrating cells produced IL-17A with approximately 50% of GM-CSF and IL-22 co-expression (Supplemental Fig. 2G).

We next sought to validate the functional activity of the in vivo reported AHR pathway expression. For this, we isolated dLNs and spinal cord cells of MOG-CFA-immunized mice and reactivated these cells with MOG peptide upon FICZ stimulation. In agreement with the *Ahr* expression pattern, the presence of FICZ resulted in considerable enhancement of IL-22 production in dLNs cells isolated at day 7 post-immunization and in CNS-infiltrating cells (Fig. 4b). Although it has been suggested that AHR activation drives IL-17A production in in vivo-generated T_H_17 cells to consequently increase autoimmune pathology, we did not observe IL-17A promotion under these conditions (data not shown). Altogether, these observations support that AHR is still expressed in CNS-infiltrating T CD4^+^ cells with encephalitogenic potential.

**Fig. 5:**
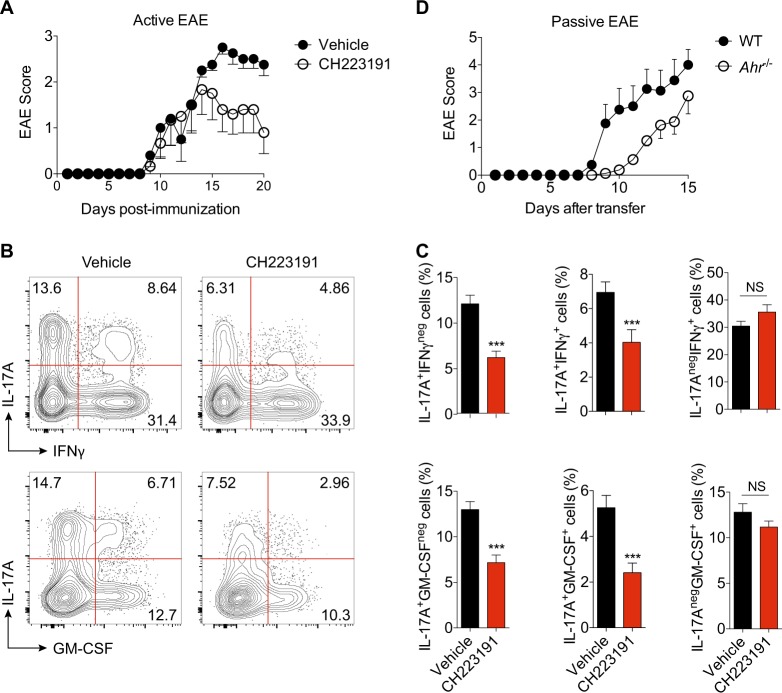
AHR activation promotes effector functions to encephalitogenic T_H_17 cells. **a** EAE clinical score of WT mice treated with AHR antagonist for 10 consecutive days (CH223191, i.p. 10 mg/kg). **b** Representative plots of IL-17A^+^, IFN-γ, and GM-CSF^+^ CNS-derived CD4^+^ T cells from vehicle and CH223191-treated EAE animals. **c** Frequency of cytokine-producer cells shown in **b**. **d** EAE clinical score of *Rag1*^−^^/−^ mice recipients of WT or *Ahr*^−^^/−^ MOG-specific T_H_17 cells. NS not significant; ***P < 0.001 (unpaired, two-tailed Student’s *t* test)

To assess the functional relevance of AHR regulation during the T_H_17 cell-mediated autoimmunity, we further inhibited AHR activity during the development of EAE by using a specific antagonist (CH223191). Inhibition of AHR during the disease course significantly impaired EAE development, which reinforces AHR participation in T_H_17-driven inflammation (Fig. 5a). We further investigated whether the EAE resistance observed in the CH223191-treated animals was associated with a defect in cytokine production from T cells. Consistent with the EAE clinical score, at peak disease, CNS-infiltrating CD4^+^ T cells from AHR-blocked mice revealed a significant defect in the expression of IL-17A, as well as in the coexpression of IL-17A with IFN-γ and GM-CSF (Fig. 5b, c). The AHR inhibition during EAE course also negatively impacted on the frequency of total GM-CSF^+^ cells, whereas IFN-γ levels were unaffected (Fig. 5b, c).

To exclude the AHR modulation in others cells by the systemic treatment with CH223191 and since AHR activation also participate during T_H_17 generation, we next analyzed the in vivo pathogenicity and effector functions of fully differentiated AHR-deficient T_H_17 cells by using a model of passive transfer of EAE. We immunized WT and *Ahr*^−^^/−^ mice with MOG (35–55) and reactivated lymphocytes from these mice in vitro with MOG peptide in the presence of IL-23 to skew the population expansion of pathogenic T_H_17 cells. AHR-deficient T cells reactivated under T_H_17-favoring conditions had significantly less ability to induce EAE after transfer into host mice, suggesting that AHR activation is important to maintain the encephalitogenic properties during late stages of T_H_17 development.

## Discussion

The idea that T_H_17 cells can be defined as pathogenic and nonpathogenic subsets came mostly from in vitro studies where isolated T CD4^+^ cells are cultured in the presence of different cytokine combinations, which may mislead the data interpretation. The AHR is highly expressed in in vitro-generated nonpathogenic T_H_17 cells; however, its activation is related to higher IL-17A/IL-22 production and exacerbation of in vivo T_H_17 cell-mediated autoimmunity^11^. Thus it remains unclear how AHR modulation impacts on the generation of subpopulations of T_H_17 cells.

By performing a series of experiments showing both the expression pattern and AHR responsiveness of different subsets of in vitro-polarized T_H_17 cells, we demonstrated that *Ahr/Cyp1a1* genes were upregulated under both pathogenic and nonpathogenic T_H_17 cell-polarizing conditions. However, while AHR was highly expressed throughout the nonpathogenic differentiation, pathogenic T_H_17 subsets expressed modest and transient levels of *Ahr*/*Cyp1a1* transcripts. As result of the shorter AHR activity in pathogenic T_H_17 cells, the AHRR—another AHR target gene—was quickly shut off in this condition.

TGFβ1 has been described as an important player for inducing *Ahr* expression in CD4^+^ T cells^12,21,30^. While previous studies did not identify IL-6 as an AHR inducer, we have observed a modest, however, significant AHR induction^12,30^. Quintana and colleagues (2008) stimulated naive T cells with different cytokine combinations and analyzed AHR expression after 4 days^12^. Kimura’s study evaluated *Ahr* transcription after 48 h, by using a different experimental approach, microarray analysis. In this latter case, it is important to note that TGFβ1 by itself presented similar effect with IL-6 stimulation^30^. Based on the kinetics experiment showed in our study, the peak of *Ahr* transcription is 24 h and it is followed by the rapid gene downregulation under IL-1β, IL-6, and IL-23 polarization (or TGFβ3+IL-6 condition). Similar profile of AHR expression was also observed when the CD4^+^ cells were stimulated with IL-6 alone (data not shown). Thus the most reasonable explanation for these differences is the time point examined in the studies.

Although TGFβ1 is as a poor *Ahr* inducer, it is clear that IL-6 downstream signaling presents a critical synergic role driving AHR gene expression. We extended these studies and demonstrated that optimal and sustained AHR expression occurs in both pathogenic and nonpathogenic T_H_17 cells when TGFβ1 is present. Interestingly, the combination of TGFβ1, but not TGFβ3, and IL-6 induced high *Ahr* expression. Although both TGFβ isoforms induce signaling through the TβRII receptor, TGFβ1 preferentially induces Smad2/3 phosphorylation^15^. Thus these data raise the possibility that the TGFβ1/Smad2/3 pathway is responsible for inducing high and sustained AHR expression during T_H_17 cell differentiation. However, further studies are necessary to address this subject.

We next observed that AHR seems to activate distinct pathways to promote IL-17A and IL-22 production by T_H_17 cells. Confirming previous studies^31,32^, we demonstrated that IL-22 production can be regulated by AHR-dependent and -independent mechanisms. Indeed, although to a lesser extent than TGFβ1-induced T_H_17 cells, cells cultured without TGFβ1 were able to enhance IL-22, but not IL-17A, upon FICZ stimulation. Whether high *Ahr* expression driven by TGFβ1 is necessary for AHR to promote *Il17a* transactivation or it indirectly regulates other AHR-associated molecules is not clear at this stage.

TGFβ1 signaling had a dual role not only by inducing optimal AHR pathway expression (and more T_H_17 cell differentiation) but also by inhibiting a T_H_17 pathogenic phenotype acquisition. Thus nonpathogenic T_H_17 subpopulation re-cultured with IL-23 had, as expected, a downregulation of *Ahr* expression and no promotion of IL-17A-induced by AHR activation. They had increased expression of the T_H_1-like pathogenicity marker T-bet (*Tbx21*). These anti-inflammatory mechanisms mediated by TGFβ1 seem to be AHR independent, since AHR-deficient T_H_17 cells did not increase pathogenic features and expressed lower levels of cytokines when compared to their AHR-sufficient counterparts. Taken together, these findings suggested that TGFβ1 signaling is crucial to maintain high *Ahr* expression and, at the same time, it opposes IL-23-driven pathogenic T_H_17 cell conversion.

By performing in vivo experiments, we were able to investigate how the AHR expression pattern is regulated on T CD4^+^ cells during a T_H_17-mediated autoimmune inflammation. Consistent with its deleterious role, we observed that AHR was positively modulated in CD4^+^ T cells during the course of EAE. CNS-infiltrating pathogenic T cells from these animals had high *Ahr/Cyp1a1* expression and enhanced IL-22 production upon AHR activation during ex vivo MOG recall. Although we and others have suggested that IL-17A is an AHR downstream effector molecule during autoimmune tissue inflammation^11,14,33^, we did not find enhancement of IL-17A during our MOG recall assay.

In accordance to published data, we further demonstrated that AHR inhibition protected mice from EAE development^11^, with a negative impact on the frequency of encephalitogenic CNS-infiltrating T_H_17 cells. Indeed, this effect was specific to AHR-deficiency in T_H_17 cells, since we observed that *Rag1*^−^^/−^ recipient mice developed milder EAE following the adoptive transfer of fully differentiated AHR-deficient IL-17A^+^ cells.

Taken together with our in vitro data, these findings suggest that, while mediators present in the microenvironment shift T_H_17 cells to either pro- or anti-inflammatory phenotype (e.g., IL-23 and TGFβ1 respectively), AHR activation would boost cytokine production in these cells^23,27^. Thus, with regards to T_H_17-mediated autoimmune diseases, the lack of AHR would impair the disease development^11,14,34^. On the other hand, when T_H_17 cells are host-protective, such as the *C. rodentium* infection, the absence of AHR signaling would negatively impact on the cytokine production of T_H_17 cells and it enhances the animals’ susceptibility^35^. In summary, the main results provided by our work unravel why and how AHR activation has been misinterpreted as an anti-inflammatory player in T_H_17 cells generated in vitro. Future studies performing fate mapping or inducible deletion of AHR activity during in vivo generation of these cells may provide useful understanding to clarify these mechanisms.

## Electronic supplementary material

Supplemental Figure 1

Supplemental Figure 2

Supplemental Table 1

Supplementary figure legends

## References

[1] Korn T, Bettelli E, Oukka M, Kuchroo VK (2009). IL-17 and Th17 Cells. Annu. Rev. Immunol..

[2] Gaffen SL, Jain R, Garg AV, Cua DJ (2014). The IL-23-IL-17 immune axis: from mechanisms to therapeutic testing. Nat. Rev. Immunol..

[3] Bettelli E (2006). Reciprocal developmental pathways for the generation of pathogenic effector TH17 and regulatory T cells. Nature.

[4] Mangan PR (2006). Transforming growth factor-beta induces development of the T(H)17 lineage. Nature.

[5] Veldhoen M, Hocking RJ, Atkins CJ, Locksley RM, Stockinger B (2006). TGFbeta in the context of an inflammatory cytokine milieu supports de novo differentiation of IL-17-producing T cells. Immunity.

[6] Ivanov II (2006). *The* orphan nuclear receptor RORgammat directs the differentiation program of proinflammatory IL-17+T helper cells. Cell.

[7] Ciofani M (2012). A validated regulatory network for Th17 cell specification. Cell.

[8] Yosef N (2013). *Dyna*mic regulatory network controlling TH17 cell differentiation. Nature.

[9] Gaublomme JT (2015). Single-cell genomics unveils critical regulators of Th17 cell pathogenicity. Cell.

[10] Veldhoen M, Hirota K, Christensen J, O’Garra A, Stockinger B (2009). Natural agonists for aryl hydrocarbon receptor in culture medium are essential for optimal differentiation of Th17 T cells. J. Exp. Med..

[11] Veldhoen M (2008). The aryl hydrocarbon receptor links TH17-cell-mediated autoimmunity to environmental toxins. Nature.

[12] Quintana FJ (2008). Control of T(reg) and T(H)17 cell differentiation by the aryl hydrocarbon receptor. Nature.

[13] Duarte JH, Di Meglio P, Hirota K, Ahlfors H, Stockinger B (2013). Differential influences of the aryl hydrocarbon receptor on Th17 mediated responses in vitro and in vivo. PLoS ONE.

[14] Nakahama T (2011). Aryl hydrocarbon receptor deficiency in T cells suppresses the development of collagen-induced arthritis. Proc. Natl. Acad. Sci. USA.

[15] Lee Y (2012). Induction and molecular signature of pathogenic TH17 cells. Nat. Immunol..

[16] Ghoreschi K (2010). Generation of pathogenic T(H)17 cells in the absence of TGF-β signalling. Nature.

[17] Wang C (2015). *CD5L/AIM* regulates lipid biosynthesis and restrains Th17 cell pathogenicity. Cell.

[18] Chalmin F (2012). Stat3 and Gfi-1 transcription factors control Th17 cell immunosuppressive activity via the regulation of ectonucleotidase expression. Immunity.

[19] McGeachy MJ (2009). The interleukin 23 receptor is essential for the terminal differentiation of interleukin 17-producing effector T helper cells in vivo. Nat. Immunol..

[20] Stockinger Brigitta, Omenetti Sara (2017). The dichotomous nature of T helper 17 cells. Nature Reviews Immunology.

[21] Apetoh L (2010). The aryl hydrocarbon receptor interacts with c-Maf to promote the differentiation of type 1 regulatory T cells induced by IL-27. Nat. Immunol..

[22] Gandhi R (2010). Activation of the aryl hydrocarbon receptor induces human type 1 regulatory T cell-like and Foxp3(+) regulatory T cells. Nat. Immunol..

[23] Gagliani N (2015). Th17 cells transdifferentiate into regulatory T cells during resolution of inflammation. Nature.

[24] Mascanfroni ID (2015). Metabolic control of type 1 regulatory T cell differentiation by AHR and HIF1-α. Nat. Med..

[25] Lee YK (2009). Late developmental plasticity in the T helper 17 lineage. Immunity.

[26] McGeachy MJ (2011). GM-CSF: the secret weapon in the T(H)17 arsenal. Nat. Immunol..

[27] Hirota K (2011). Fate mapping of IL-17-producing T cells in inflammatory responses. Nat. Immunol..

[28] Yang Y (2009). T-bet is essential for encephalitogenicity of both Th1 and Th17 cells. J. Exp. Med..

[29] Heink S (2017). Trans-presentation of IL-6 by dendritic cells is required for the priming of pathogenic TH17 cells. Nat. Immunol..

[30] Kimura A, Naka T, Nohara K, Fujii-Kuriyama Y, Kishimoto T (2008). Aryl hydrocarbon receptor regulates Stat1 activation and participates in the development of Th17 cells. Proc. Natl. Acad. Sci. USA.

[31] Yeste A (2014). IL-21 induces IL-22 production in CD4+T cells. Nat. Commun..

[32] Rutz S (2011). Transcription factor c-Maf mediates the TGF-β-dependent suppression of IL-22 production in T(H)17 cells. Nat. Immunol..

[33] Veldhoen M (2017). Interleukin 17 is a chief orchestrator of immunity. Nat. Immunol..

[34] Talbot J (2018). Smoking-induced aggravation of experimental arthritis is dependent of aryl hydrocarbon receptor activation in Th17 cells. Arthritis Res. Ther..

[35] Schiering C (2017). Feedback control of AHR signalling regulates intestinal immunity. Nature.

