# Supplementary figures and images for: TGFβ1 signaling sustains aryl hydrocarbon receptor (AHR) expression and restrains the pathogenic potential of TH17 cells by an AHR-independent mechanism
Source: Cell Death Dis. 2018 Nov 13;9(11):1130. doi: 10.1038/s41419-018-1107-7 (PMC6234206; doi:10.1038/s41419-018-1107-7)

# Supplemental Figure 1

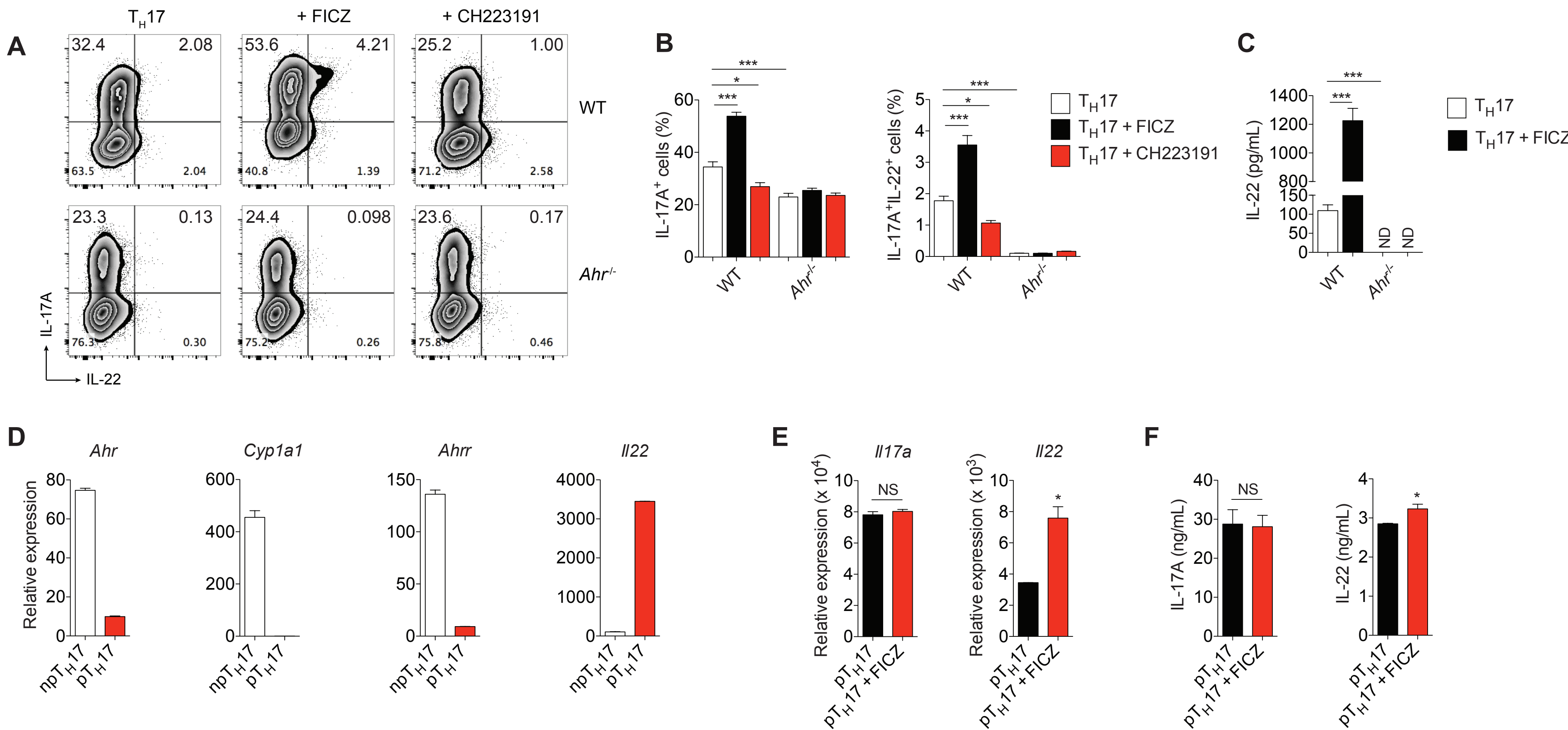

Supplement: Supplementary file 1 — Supplemental Figure 1 [file 41419_2018_1107_MOESM1_ESM.pdf]

Supplemental Figure 2

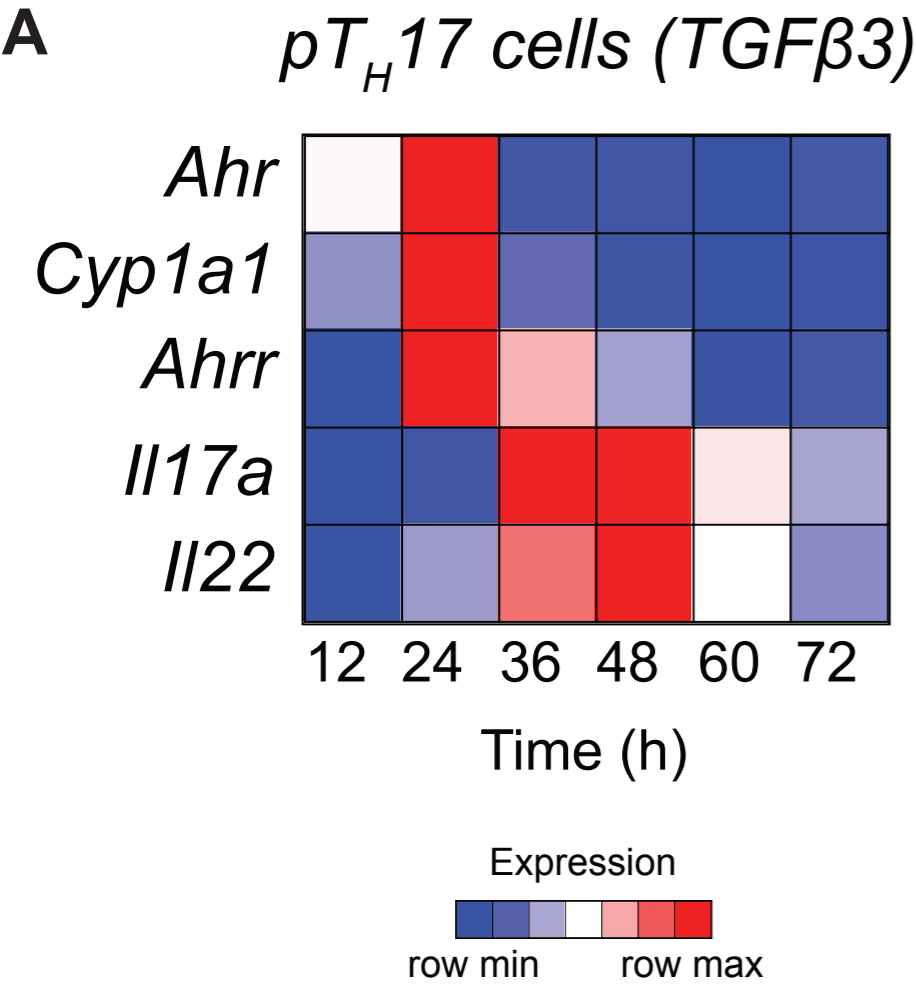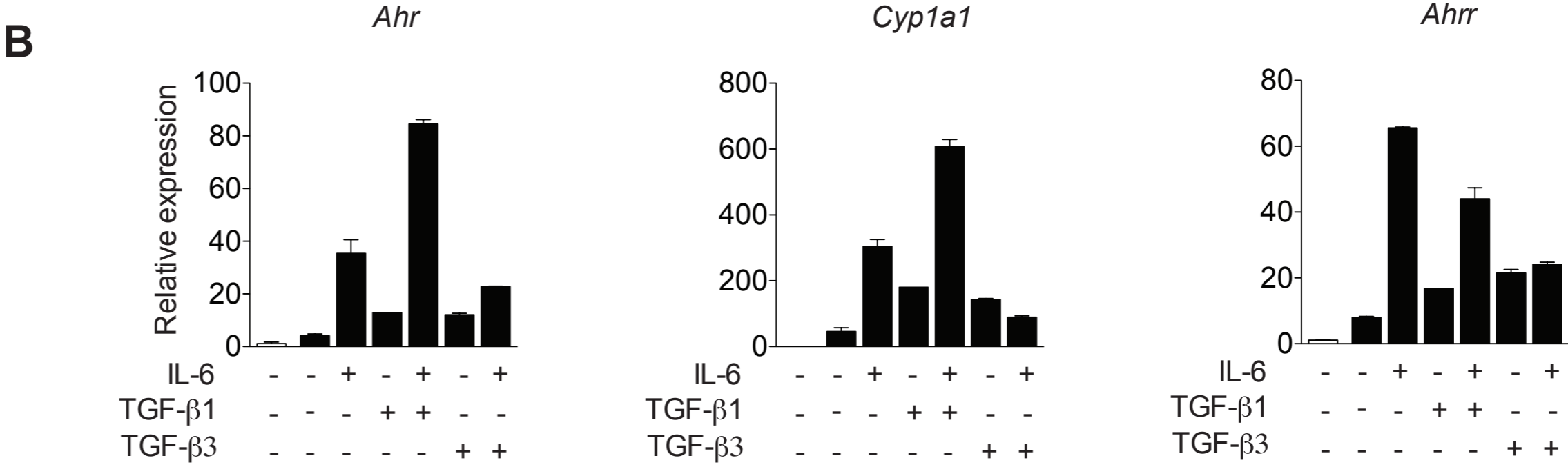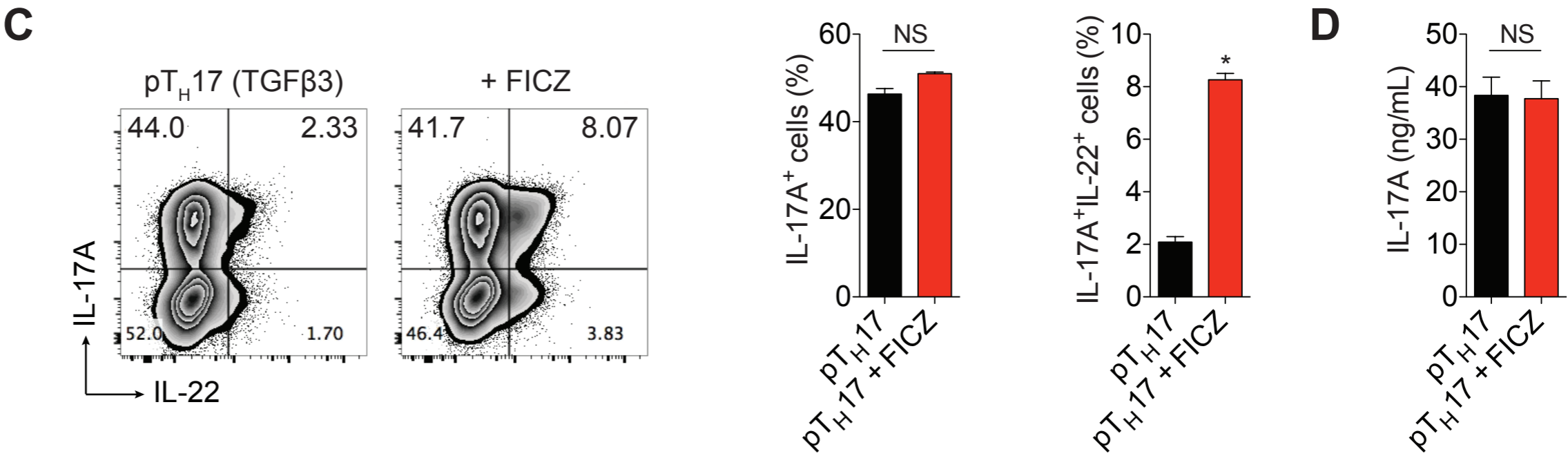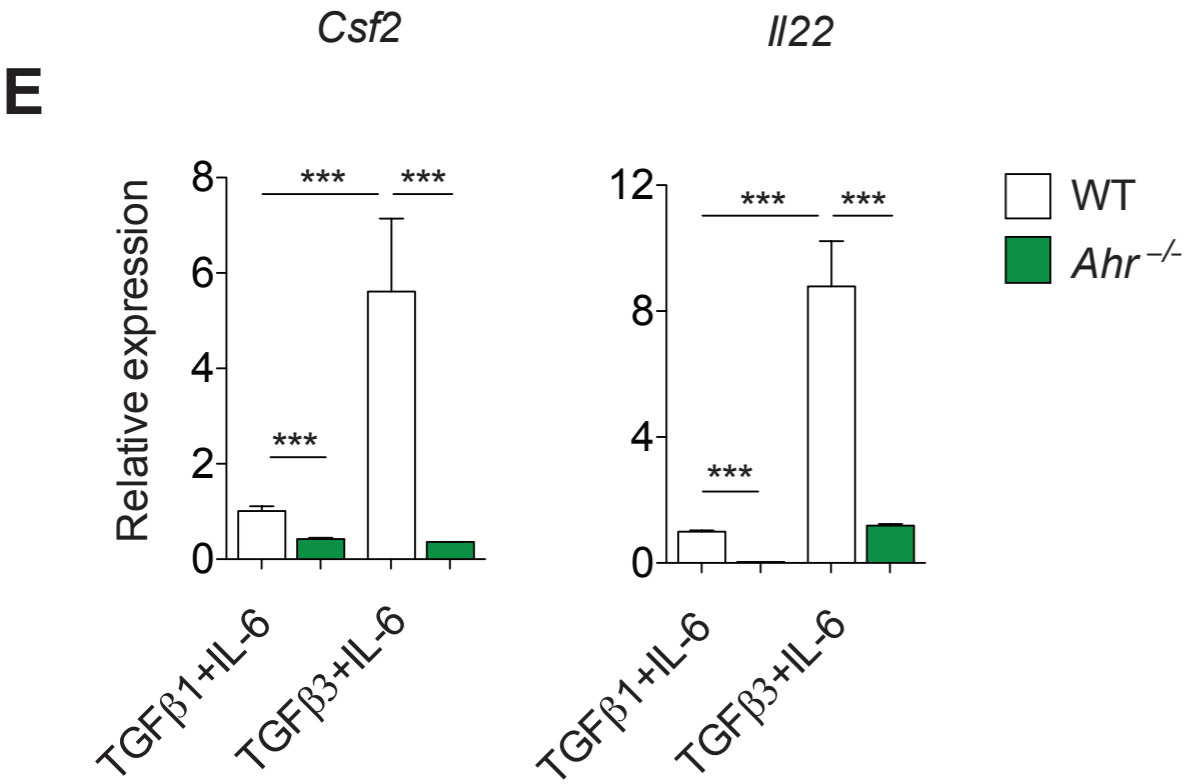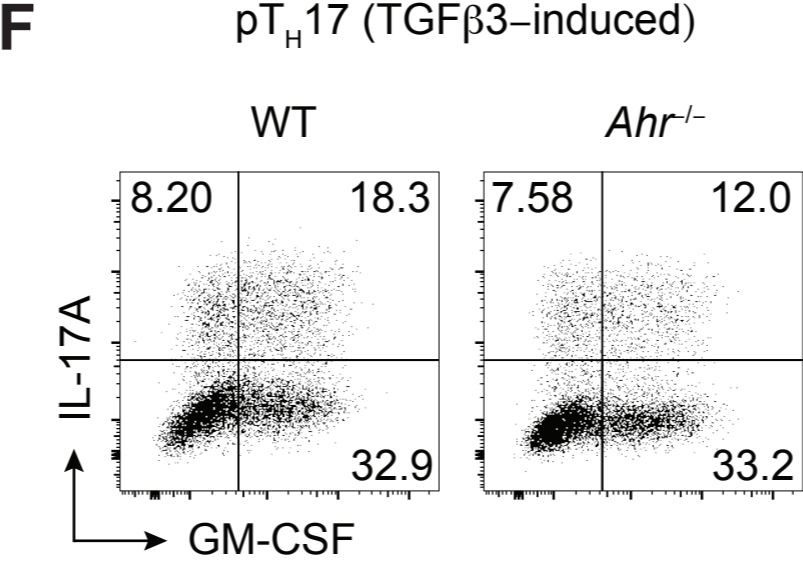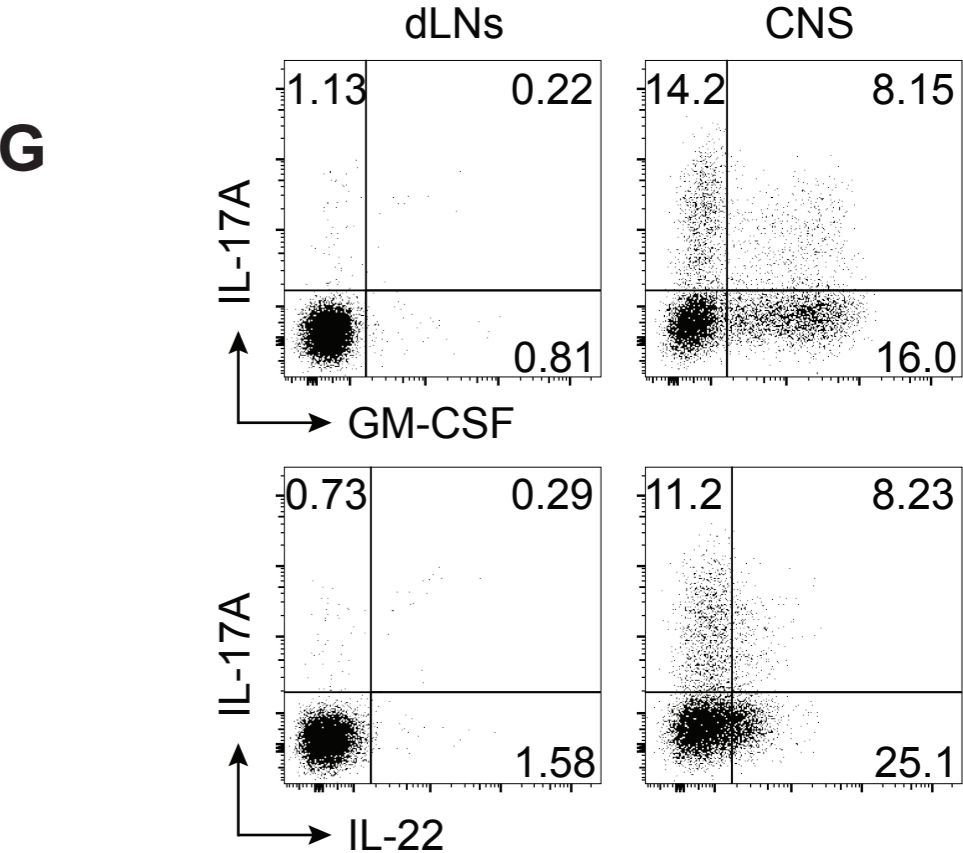

Supplement: Supplementary file 2 — Supplemental Figure 2 [file 41419_2018_1107_MOESM2_ESM.pdf]
