# Supplementary material for: TGFβ1 signaling sustains aryl hydrocarbon receptor (AHR) expression and restrains the pathogenic potential of TH17 cells by an AHR-independent mechanism
Source: Cell Death Dis. 2018 Nov 13;9(11):1130. doi: 10.1038/s41419-018-1107-7 (PMC6234206; doi:10.1038/s41419-018-1107-7)
Supplement: Supplementary file 3 — Supplemental Table 1 [file 41419_2018_1107_MOESM3_ESM.pdf]

SUPPLEMENTAL TABLE 1

TGFβ1 + IL-6						
Gene/Time (h)	12	24	36	48	60	72
Ahr	21.09	55.8	64.71	68.26	74.68	49.97
Cyp1a1	56.76	724.84	1115.84	541.37	455.17	438.07
Ahrr	3.4	29.74	77.59	79.59	135.96	187.41
Il22	6.9	26.04	64.29	69.09	105.32	109.29
Il17a	3.31	115.91	1250.66	3764.82	5457.52	6712.19

IL-1β + IL-6 + IL-23						
Gene/Time (h)	12	24	36	48	60	72
Ahr	9.11	23.13	9.75	9.27	3.87	4.95
Cyp1a1	44.01	246.77	63.23	8.13	1.01	1.85
Ahrr	2.21	52.51	59.2	30.47	8.8	8.05
Il22	1.02	101.93	742.72	1367.23	912.45	777.72
Il17a	56.85	1714.3	3079.22	46747.07	78105.3	129910.6

TGFβ3 + IL-6						
Gene/Time (h)	12	24	36	48	60	72
Ahr	7.71	24.77	2.82	2.67	1.90	3.01
Cyp1a1	31.36	250.71	22.48	5.82	0.86	5.52
Ahrr	2.21	40.82	21.46	9.51	2.82	4.63
Il22	5.04	102.01	261.84	341.91	162.85	93.78
Il17a	106.57	495.72	6017.76	5769.34	3256.39	1957.86
