# Supplementary material for: TGFβ1 signaling sustains aryl hydrocarbon receptor (AHR) expression and restrains the pathogenic potential of TH17 cells by an AHR-independent mechanism
Source: Cell Death Dis. 2018 Nov 13;9(11):1130. doi: 10.1038/s41419-018-1107-7 (PMC6234206; doi:10.1038/s41419-018-1107-7)
Supplement: Supplementary file 4 — Supplementary figure legends [file 41419_2018_1107_MOESM4_ESM.docx]

**SUPPLEMENTAL FIGURE 1. AHR regulates IL-17A and IL-22 production in nonpathogenic T_H_17 cells.** (**A**) Representative plots of IL-17A^+^ and IL-22^+^ cells from wild-type (WT) and *Ahr*^-/-^ mice. T_H_17 cells were differentiated with TGFβ1 plus IL-6 in the presence of Vehicle (DMSO), the AHR agonist FICZ (100 nM) or the AHR antagonist CH223191 (3 µM) and assessed by flow cytometry. (**B**) Frequency of IL-17A^+^ (left) and IL-17A^+^IL-22^+^ (at the right) showed in **A**. (**C**) Enzyme-linked immunosorbent assay of IL-22 in supernatants of T_H_17 cells differentiated from WT and *Ahr*^-/-^ mice under FICZ stimulation. (**D**) Quantitative RT-PCR analysis of *Ahr*, *Cyp1a1*, *Ahrr*, and *Il22* mRNA in CD4^+^ naïve T cells differentiated for 60 h under nonpathogenic (npT_H_17, TGFβ1 plus IL-6, in white bars) and pathogenic T_H_17 cells (pT_H_17, IL-1β, IL-6 and IL-23, in red bars) conditions; results were calculated by the comparative threshold cycle method and are presented relative to those of *Gapdh* (internal control gene encoding glyceraldehyde phosphate dehydrogenase). Pathogenic T_H_17 cells were generated as in **D** and AHR was activated by FICZ stimulation. (**E**) qPCR analysis of *Il17* and *Il22* and (**F**) ELISA of IL-17A and IL-22 in the culture supernatants. ND, not determined; NS, not significant, **P* < 0.05, and ****P* < 0.001 (**B** and **C**, two-way analysis of variance (ANOVA); **E** and **F**, unpaired, two-tailed Student’s *t*-test). Data are representative of more than three independent experiments with similar results.

**SUPPLEMENTAL FIGURE 2. AHR is activated during TGFβ3-induced pathogenic T_H_17-cell differentiation and it regulates IL-22 production.** (**A**) Heatmap of *Ahr*, *Cyp1a1* and *Ahrr* mRNA expression in pathogenic T_H_17 cells generated from naïve CD4^+^ T cells for 12, 24, 36, 48, 60, and 72 h with TGFβ3 plus IL-6. (**B**) qPCR analysis of *Ahr*, *Cyp1a1* and *Ahrr* mRNA expression of naïve CD4^+^ T cells activated 24 h under different combinations of IL-6, TGFβ1, and TGFβ3. (**C**) Frequency of IL-17A^+^ and IL-22^+^ cells from pT_H_17 cells (TGFβ3) differentiated in the presence of FICZ. (**D**) ELISA of IL-17A in the culture supernatants of cells polarized as shown in **C**. (**E**) qPCR analysis of *Csf2* and *Il22* of wild-type (WT, white bars) and *Ahr*-deficient (green bars) naive CD4^+^ T cells differentiated for 72 h with TGFβ1 + IL-6 or TGFβ3 + IL-6. (**F**) Frequency of IL-17A^+^ and GM-CSF^+^ cells from wild-type (WT) and *Ahr*-deficient naive CD4^+^ T cells differentiated as shown in **F**. (**G**) Representative IL-17A^+^, GM-CSF^+^, and IL-22^+^ cells from the draining lymph nodes (dLNs, left) and spinal cord (CNS, right) 14 days after EAE immunization. NS, not significant; **P* < 0.05 and ****P* < 0.001 (unpaired, two-tailed Student’s *t*-test). Data are representative of more than three independent experiments with similar results.

**SUPPLEMENTAL TABLE 1. AHR signaling pathway is transiently expressed in T_H_17 cells generated by IL-1β, IL-6, IL-23 and TGFβ3 plus IL-6.** (**A**) Fold induction of *Ahr,* *Cyp1a1,* *Ahrr*, *Il22*, and *Il17a* mRNA expression in CD4^+^CD44^lo^CD62L^hi^ naïve T cells differentiated for 12, 24, 36, 48, 60, and 72 h under nonpathogenic (TGFβ1 plus IL-6) and pathogenic (IL-1β, IL-6 and IL-23 or TGFβ3 plus IL-6) T_H_17 conditions. Fold induction is relative to the normalized gene expression of fresh naïve T CD4^+^ cells isolated for each specific polarization.
